# Comparison of Traditional and Industrial Sausages Baranjski Kulen and Kulenova Seka Using Comprehensive Proteome, Peptidome and Metabolome Techniques

**DOI:** 10.17113/ftb.60.02.22.7374

**Published:** 2022-06

**Authors:** Valerija Šimunec, Rea Bertoša, Anita Šporec, Igor Lukić, Diana Nejašmić, Marija Lovrić, Ivana Dodig, Nada Vahčić, Ana Butorac

**Affiliations:** 1 Faculty of Food Technology and Biotechnology, Pierottijeva 6, 10000 Zagreb, Croatia; 2 BICRO BIOCentre, Ltd., Borongajska cesta 83 H, 10000 Zagreb, Croatia; 3 Institute of Agriculture and Tourism, Karla Huguesa 8, 52440 Poreč, Croatia; 4 Pliva Ltd., Prilaz Baruna Filipovića 25, 10000 Zagreb, Croatia

**Keywords:** industrial fermented sausage, traditional fermented sausage, baranjski kulen, kulenova seka, proteomics, peptidomics, aroma profile

## Abstract

**Research background:**

Baranjski kulen is one of the most popular fermented meat sausages originating from Croatia. It has protected geographical indication, and is traditionally produced in the Baranja region of Croatia. Kulenova seka is a fermented sausage very similar to baranjski kulen, but it has a different calibre and consequently, a shorter time of production. In recent decades, due to the high demand and popularity of these products, industrially produced baranjski kulen and kulenova seka have become available on the market. This work aims to identify specific characteristics of traditional and industrial sausages baranjski kulen and kulenova seka on proteome, peptidome and metabolome level, which could potentially lead to better optimization of the industrial production process in order to obtain an equivalent to the traditional product.

**Experimental approach:**

Protein profiles of baranjski kulen and kulenova seka (traditional and industrial) were analysed using two-dimensional gel electrophoresis followed by differential display analysis and protein identification by mass spectrometry. Peptidomic profile was analysed *via* liquid chromatography-tandem mass spectrometry. Furthermore, aroma profiles were investigated *via* headspace solid phase microextraction and gas chromatography-mass spectrometry.

**Results and conclusions:**

The major identified characteristics of each product were: industrial baranjski kulen: specific degradation of myosin-1 and titin, overabundance of stress-related proteins and increased phenylalanine degradation; traditional baranjski kulen: decreased concentration of phenylalanine and overabundance of fructose-bisphosphate aldolase A and carbonic anhydrase 3; industrial kulenova seka - specific myosin-4 and haemoglobin subunit alpha degradation process; traditional kulenova seka - overabundance of dihydropyrimidine dehydrogenase [NADP(+)] and myosin light chain 1/3, skeletal muscle isoform, degradation of albumin and myoglobin, decreased concentrations of almost all free amino acids and increased amounts of smoke-derived volatile compounds. Presented results showed that potential product type-specific quality markers for each sausage could be found.

**Novelty and scientific contribution:**

In this preliminary communication, the first insights into protein degradation processes and generation of peptides, free amino acids and aroma compounds of industrial and traditional baranjski kulen and kulenova seka are presented. Although further research is needed to draw general conclusions, the specific profile of proteins, peptides, amino acids and volatile compounds represents the first step in the industrial production of sausages that meet the characteristics of traditional flavour.

## INTRODUCTION

Traditional fermented meat production has a long history and is very popular in different parts of the world. Variations in production conditions often contribute to specificity in texture and flavour (*1*, *2*). Due to the high demand and popularity of fermented meat products, small traditional production is often replaced by large industrial production. However, obtaining a final product that meets the texture and flavour of the traditional product is still a challenge for the meat industry (*3*).

Attributes of fermented meat products are derived from extensive protein changes occurring during fermentation and maturation processes. Proteolysis is one of the most important biochemical changes that influences both texture and flavour development due to the formation of several low-molecular-mass compounds, including peptides, free amino acids, aldehydes, organic acids and amines (*4*). Generally, it is known that hydrolysis of meat proteins generates polypeptides by endogenous muscle enzymes, such as cathepsins, calpains and proteasome (*5*, *6*). The polypeptides can be further degraded to smaller peptides and free amino acids by the action of peptidases and aminopeptidases from both muscle and microorganisms (*2*, *7*). Free amino acids released during ripening directly contribute to the basic taste of dry fermented sausages. Amino acids are precursors of many volatile compounds thus indirectly contributing to the development of the typical aroma of the final meat product (*2*, *5*). It is well known that technological conditions during fermentation may determine and/or regulate protein degradation (*8*). Furthermore, in order to shorten the ripening time, ensure colour development, enhance flavour, improve product safety, and preserve the original sensory quality of the products, starter cultures are frequently used in industrial production (*1*).

Baranjski kulen is a fermented sausage with protected geographical indication (PGI), and it is traditionally produced in Baranja region of Croatia. Traditional baranjski kulen is a naturally fermented sausage made with minced pork meat and pork back fat, mixed with spices and filled in a natural casing, derived from a pig’s caecum. Kulenova seka is a fermented sausage very similar to baranjski kulen but made by stuffing the meat into a natural casing derived from pig’s rectum. These two types of sausages have very similar raw material; however, they are different in calibre, and consequently, time of production. Currently, the information about the similarity of baranjski kulen and kulenova seka on the molecular level is still limited.

This preliminary communication presents the first insights into protein degradation processes and generation of peptides, free amino acids, and aroma compounds of industrial and traditional baranjski kulen and kulenova seka. Data obtained for all products are compared, and the results enabled identification of specific characteristics of each product.

## MATERIALS AND METHODS

### Sausage sampling

Baranjski kulen and kulenova seka were purchased from the Matijević family farm (Suza, Croatia) and from the Croatian meat industry Belje plus Ltd. (Beli Manastir, Croatia) in 2016. The production process of baranjski kulen (PGI) is precisely specified and very similar to the production process of kulenova seka (*9*). A brief description of the production of both products with an emphasis on the differences between baranjski kulen and kulenova seka as well as the differences in the process between traditionally and industrially produced sausages is given below.

Raw materials for production of these sausages originated from T2 (pigs with higher final mass) and K (sows obtained by breeding) category pigs. For traditional sausages, the raw material was obtained only from the T2 category, while both T2 and K category pigs were used for the industrial version. At least 80% of the meat part must come from the first category meat (ham and loin), while the use of third category meat is not allowed. According to a specified process, prior to preparation of the filling, the raw material was tempered, meat at between -2 and 4 °C and back fat at between  -8 and 4 °C. The tempered and pre-minced back fat was then mixed with the meat in a required meat/fat ratio (in kg/kg: 90:10 for baranjski kulen and 80:20 for kulenova seka) with the addition of sweet red pepper, red hot pepper, ground white pepper, garlic and salt. To the best of our knowledge, in traditional sausages fresh garlic and sucrose were added without other additives. On the other hand, in industrial sausages, besides dry garlic and dextrose, starter cultures (*Staphylococcus equorum* and *Lactobacillus curvatus*) were added for fermentation control along with antioxidants, nitrites and nitrates. At this point, the mixture was minced to a granulation of 6 or 8 mm and filled into pork intestines. Baranjski kulen is filled in a natural casing, derived from pig’s caecum, while kulenova seka is filled in a natural casing derived from pig’s rectum. Prior to fermentation, conditioning was carried out for 24 h. Sausages were then transferred into the fermentation chamber for at least 7 days. During fermentation, the products were periodically smoked, provided that the smoke temperature did not exceed 22 °C. Fermentation conditions such as temperature (5-24 °C), air humidity (75-92%) and air circulation were monitored only in industrial processing. The ripening lasted for at least three months at a temperature up to 15 °C and air humidity from 60 to 85%. The main differences from traditional products were in the duration and control of fermentation and ripening conditions. Consequently, the total processing time for industrial baranjski kulen was 90 days, industrial kulenova seka 50 days, traditional baranjski kulen 150 days, and for traditional kulenova seka 65 days. For the purpose of preliminary investigation, one of each industrially and traditionally produced baranjski kulen and kulenova seka were analysed.

### Protein, peptide and amino acid isolation from sausages

A representative portion of each sausage was homogenized in distilled water for 5 min (1:4, g/g). The supernatant was decanted, filtered and then centrifuged (5415R; Eppendorf, Hamburg, Germany) at 10 000×*g* and 4 °C for 15 min. The supernatant was placed in a clean tube and used for soluble protein fraction, peptide and amino acid analyses. One of each industrially and traditionally produced baranjski kulen and kulenova seka were analysed. All samples were prepared in duplicate.

### Protein analysis

Two-dimensional gel electrophoresis (2-DE) was performed as described by Butorac *et al*. (*10*). Isoelectric focusing (IEF) was performed using Flatbed IEF System (Cleaver Scientific, Rugby, UK). The second dimension, sodium dodecyl sulphate–polyacrylamide gel electrophoresis (SDS-PAGE) was run with OmniPAGE System (Cleaver Scientific) through 1-mm thick 12% polyacrylamide gel. The proteins were visualized by Coomassie Brilliant blue staining. 2-DE gels were made in duplicates. All chemicals and materials were purchased from Sigma-Aldrich Co., Merck, Darmstadt, Germany. Densitometry analysis was performed with image analysis software (Discovery Series PDQuest 2-DE analysis software, v. 7.4.0) (*11*) integrated with a VersaDoc 4 000 Imaging System (Bio-Rad, Hercules, CA, USA). Master gel was created automatically based on the differentially expressed spots identified across all the gels. Differential analysis was obtained by overlapping images of gels based on Gaussian distribution with a cut-off>3.0.

Differentially displayed spots were excised from gels and subjected to tryptic in-gel digestion according to the procedure described by Shevchenko *et al*. (*12*). After digestion, the extracted peptides were purified with reversed-phase columns (ZipTip C4; Millipore, Darmstadt, Germany) and 0.5 µL of purified peptides was spotted onto a metal matrix-assisted laser desorption/ionization (MALDI) plate. Sample droplets were dried, and then 0.5 µL of matrix solution was added. The matrix contained 1.4 mg of α-cyano-4-hydroxycinnamic acid (CHCA; Sigma-Aldrich, Merck) dissolved in 1 mL of solvent mixture containing 85% acetonitrile (ACN) and 15% aqueous solution of 0.1% trifluoroacetic acid (TFA).

Mass spectrometry (MS) was acquired with MALDI time-of-flight/time-of-flight (TOF/TOF) (Autoflex Speed; Bruker, Bremen, Germany) in a positive ion reflection mode. The instrument parameters were set using Flex Control v. 3.4 software (Bruker) (*13*). Mass spectra were obtained by averaging 1000 laser shots covering a mass range *m/z*=700-4000. After the recording of MS spectra, ten of the most intense precursor signals were selected for tandem mass spectrometry analysis (MS/MS). Protein identification and database search were performed by ProteinScape v. 3.1.0 software (Bruker) (*14*). Combined MS and MS/MS data were matched against the SwissProt database (*15*) by MASCOT search engine. The parameters were set as follows: one trypsin miscleavage, oxidation of methionine and 100 ppm precursor mass tolerance. The Gene Ontology (GO) Consortium’s online tool (http://www.geneontology.org/) was used for functional characterization of differentially expressed proteins (*16*).

### Peptide analysis

The volume of 500 µL of each sample prepared as described in the section *Protein, peptide and amino acid isolation from sausages* was pipetted in Microcon filter 10 kDa (Millipore, Bedford, MA, USA) and was several times recentrifuged at 5000×*g* (5415R; Eppendorf) until 200 µL of the filtrate were collected.

The NanoLC system Dionex Ultimate 3 000 RSLCnano (Thermo Fisher Scientific, Waltham, MA, USA) equipped with a UV/Vis detector coupled to Proteineer fcII (Bruker) was used for peptide separation and collection directly onto the MALDI plate. Chromatographic separation was performed on a column Acclaim PepMap 100 C18 3 µm, 100 Å, 75 µm i.d.×15 cm (Thermo Fisher Scientific) at 40 °C. The flow rate was 0.3 µL/min, and the injection volume was 1 µL. Mobile phase A consisted of 0.1% aqueous solution of TFA (*V*/*V*) and mobile phase B consisted of 0.1% TFA in ACN. The 75-minute gradient elution was programmed to increase over 70 min with solvent B from 2 to 90% and then to condition the column back to the initial conditions until the completion of the run. The spotter make-up flow was set to 100 µL/h (1.4 mg CHCA matrix dissolved in 1 mL of 50% ACN aqueous solution). The total number of collected fractions was 192.

MS acquisition was performed using MALDI-TOF/TOF (Autoflex Speed; Bruker). Acquisition was performed in positive ion reflection mode. The instrument parameters were set using Flex Control v. 3.4 software (Bruker) (*13*). Mass spectra were obtained by averaging 1000 laser shots covering a mass range *m/z*=700-4 000. MS/MS analysis was done with the following parameters: signal-to-noise (S/N) threshold 10, 100 ppm mass tolerance between compounds, merging compounds separated by less than 6 fractions, 5.0 Da as minimal mass distance to co-eluting compounds. Using the SwissProt protein database (*15*), mammal taxonomy was searched with ProteinScape v. 3.1.0 (Bruker) (*14*). A search using MS/MS data was matched against the Swiss-Prot database (*15*) by the MASCOT search engine. The parameters were set as follows: unspecified cleavage, oxidation on methionine, asparagine and glutamine deamination as variable modifications and 100 ppm mass tolerance. Functional characterization (GO) of identified proteins was performed by the above-mentioned online tool (*16*).

### Free amino acid analysis

The volume of 500 µL of each sample was prepared as described above for peptide analysis, the difference being in pipetting in Microcon filter 3 kDa (Millipore, Bedford). Then it was recentrifuged several times at 5000×*g* (Eppendorf) until 200 µL of filtrate were collected. Filtrate was diluted 5 times in water, and 10 µL of diluted sample were derivatized with AccQ-Tag reagent (Waters, Milford, MA, USA) according to the manufacturer’s instructions.

Amino acid analysis was performed as described in Waters AccQ-Tag Chemistry Package instruction manual using a Waters liquid chromatography system with fluorescent detector. Individual amino acids were identified by comparison of their retention times with those of calibration standards.

### Aroma profiling

Volatile extraction by headspace solid phase microextraction (HS-SPME) was carried out according to the modified method of Ma *et al*. (*17*). Representative sausage samples were minced in saturated sodium chloride solution (1:5, g/g). The solution was mixed at room temperature for 20 min. An aliquot (10 mL) of homogenized sample was placed in 20-mL flat bottom headspace vial along with the magnet and then capped with a crimp cap fitted with a polytetrafluoroethylene (PTFE)/silicone septum (Supelco, Bellefonte, PA, USA). The headspace vial was heated at 40 °C for 30 min in order to achieve equilibration. SPME carboxen/polydimethylsiloxane (PDMS) fibre (Agilent Technologies, Santa Clara, CA, USA) was preconditioned prior to analysis at 250 °C for 10 min in a GC injection port. Then, the volatile components in the samples were adsorbed onto the fibre for 180 min. One of each industrially and traditionally produced baranjski kulen and kulenova seka were analysed. All samples were prepared in duplicate.

After extraction, the SPME fibre was inserted directly into the injection port of the gas chromatograph GC 7890B equipped with 7693A ALS interface and 240 Ion Trap GC/MS detector (Agilent Technologies). The volatile compounds were immediately thermally desorbed for 2 min at 250 °C in the SPME-specific liner in the injector port of GC-MS. A DB-35MS capillary column (35%-phenyl)-methylpolysiloxane phase, 30 m×0.25 mm×0.25 µm (Agilent Technologies) was used for separation. Helium 6.0 (purity 99.9999%) was used as a carrier gas with a constant flow rate of 1.5 mL/min. Chromatographic conditions were as follows: the oven was held for 2 min at 40 °C, heated to 240 °C at 5 °C/min, and held for 3 min at this temperature. The total run time was 45 min. The mass spectrometer was operated in the electron impact mode with a source temperature of 200 °C, an ionizing voltage of 70 eV, the transfer line temperature of 240 °C, manifold temperature of 40 °C and ion trap temperature of 150 °C. The mass spectrometer scanned masses from 30 to 350 *m/z* at a scan time of 2 µscan/s and maximum ionization time 65 000 µs. Peak identification was carried out by comparing the obtained mass spectra with those in the NIST 2014/EPA 7/NIH Mass Spectral Database (*18*). The data were processed by the analysis of variance (one-way ANOVA) and the least significant difference (LSD) *post* hoc test was used to compare the mean values of concentrations at p<0.05. Statistica v. 13.2 software (*19*) was used. All four products were compared with each other based on the obtained results.

## RESULTS AND DISCUSSION

For the purpose of the preliminary study, we have analysed the industrially and traditionally produced sausages baranjski kulen and kulenova seka *via* the proteomic and peptidomic approach complemented with the amino acid analysis and aroma profile analysis. The main goal was to make comparisons between protein degradation processes and generation of peptides, free amino acids and aroma compounds to reveal product specificity.

### Proteome and peptidome analysis

**Fig. 1:**
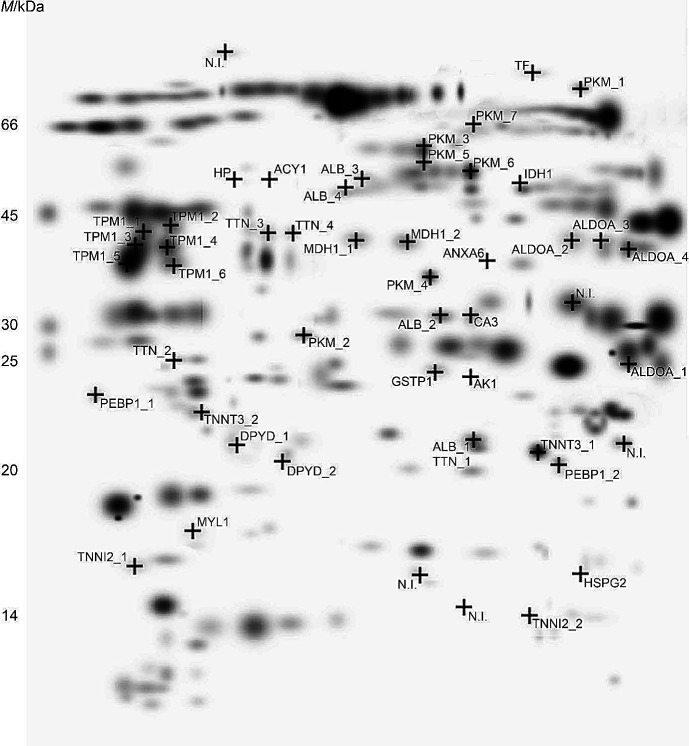
A master gel from a 2-DE gel electrophoresis showing differentially expressed soluble protein fractions isolated from industrial and traditonal baranjski kulen, and industrial and traditonal kulenova seka. Black crosses show protein spots that are differentially expressed among products. Protein spots are identified after mass spectrometry analyses and gene names are linked to each spot. Number after gene name is attached to proteins identified in more than one spot. Densitometric values of each spot and mass spectrometry results are shown in Table 1. N.I.=not identifed spot

A total of 435 protein spots were detected on 2-DE gels of industrial baranjski kulen and 450 on 2-DE gels of the traditional sample. A total of 446 protein spots were detected on 2-DE gels of industrial kulenova seka and 450 on 2-DE gels of the traditional sample. In comparative analysis of all 2-DE gels, 51 spots were detected as differentially displayed, of which 46 were successfully identified. Fig. 1 shows an image of the 2-DE master gel. Table 1 presents the complete list of significantly altered proteins when comparing all four products. Based on the data obtained from the MASCOT database, 11 proteins were identified in more than one spot. Generally, a protein shift on 2-DE gel can be associated with posttranslational modification that leads to a change in overall protein charge and/or molecular mass or can be due to limited proteolysis (*20*).

**Table 1:** Differentially expressed soluble proteins in traditional and industrial baranjski kulen and kulenova seka detected in 2-DE (Fig. 1) by matrix-assisted laser desorption/ionization time-of-flight/time-of-flight (MALDI-TOF/TOF) mass spectrometry analysis

Biological process^a^	Accession no^b^/gene	Protein name^c^	*M*_theoretical_/kDa	Spot^d^	Score^e^	*M*_estimated_/kDa	Kulenova seka		Baranjski kulen	
Industrial	Traditional	Industrial	Traditional
*A*_spot_/10 000^f^			
Small molecule metabolic process (GO:0044281)	CAH3/CA3	Carbonic anhydrase 3	29	CA3	36	30	n.d.	n.d.	n.d.	235.0±96.5
	ALDOA/ ALDOA	Fructose-bisphosphate aldolase A	40	ALDOA_1	370	25	n.d.	n.d.	n.d.	863.2±32.2
				ALDOA_2	248	40	n.d.	n.d.	n.d.	44.8±16.7
				ALDOA_3	189	40	n.d.	n.d.	n.d.	88.7±65.8
				ALDOA_4	179	40	n.d.	n.d.	n.d.	201.5±146.1
	ACY1/ ACY1	Aminoacylase-1	46	ACY1	376	46	(60.0±12.4)*	(194.5±17.4)**	n.d.	n.d.
	IDHC/ IDH1	Isocitrate dehydrogenase [NADP]	46	IDH1	135	46	(81.7±26.1)*	(298.9±25.7)**	140.0±49.7	127.8±56.0
	DPYD/ DPYD	Dihydropyrimidine dehydrogenase [NADP(+)]	111	DPYD_1	37	21	(33.4±11.2)*	(167.8±21.2)**	n.d.	n.d.
				DPYD_2	38	20	(43.4±35.0)*	(172.4±35.0)**	n.d.	n.d.
	MDHC/MDH1	Malate dehydrogenase, cytoplasmic	37	MDH1_1	368	37	n.d.	126.4±108.9	n.d.	339.5±136.7
				MDH1_2	65	37	(89.9±42.1)*	(403.7±48.6)**	n.d	(418.5±26.0)**
	KPYM/PKM	Pyruvate kinase	58	PKM_1	117	58	n.d.	n.d.	1265.7±146.6	n.d.
				PKM_2	38	27	107.1±61.8	128.2±56.4	300.4±58.7	n.d.
				PKM_3	254	50	(81.3±51.9)*	(35.0±12.3)*	n.d.	(681.2±74.5)**
				PKM_4	105	35	n.d.	n.d.	n.d.	352.7±123.9
				PKM_5	360	52	n.d.	89.3±55.7	n.d.	86.7±65.8
				PKM_6	180	55	n.d.	(685.3±100.4)*	(90.7±23.6)**	(168.9±76.4)**
				PKM_7	322	55	n.d.	(211.4±26.5)*	n.d.	(685.3±21.5)**
	KAD1/AK1	Adenylate kinase isoenzyme 1	22	AK1_1	207	24	n.d.	n.d.	193.7±74.1	n.d.
				AK1_2	64		211.8±76.0	n.d.	n.d.	500.8±221.4
Cellular response to stress (GO:0033554)	TRFE/TF	Serotransferrin	77	TF	255	60	n.d.	n.d.	(390.2±45.0)*	(100.9±19.5)**
	GSTP1/ GSTP1	Glutathione S-transferase P	25	GSTP1	97	24	n.d.	n.d.	216.5±29.1	n.d.
	ANXA6/ANXA6	Annexin A6	75	ANXA6	74	37	n.d.	n.d.	45.4±29.5	n.d.
Muscle contraction (GO:0006936)	MYL1/MYL1	Myosin light chain 1/3, skeletal muscle isoform	16	MYL1	164	16	n.d.	123.5±66.8	n.d.	n.d.
	TNNT3/ TNNT3	Troponin T, fast skeletal muscle	32	TNNT3_1	186	21	392.1±171.2	n.d.	n.d.	n.d.
				TNNT3_2	247	23	(530.1±87.3)*	(53.9±97.0)**	n.d.	(546.8±97.8)*
	TNNI2/TNNI2	Troponin I	21	TNNI2_1	77	16	652.4±72.8	(349.7±96.7)**	512.0±32.5	(1792.9±478.9)*
				TNNI2_2	125	14	n.d.	(556.8±18.4)*	n.d.	(75.5±65.2)**
	TITIN/TTN	Titin	2066	TTN_1	133	22	n.d.	n.d.	263.4±65.4	n.d.
				TTN_2	101	25	n.d.	n.d.	112.2±78.9	n.d.
				TTN_3	100	38	263.6±47.9	n.d.	n.d.	n.d.
				TTN_4	114	38	418.0±150.8	n.d.	n.d.	n.d.
	TPM1/TPM1	Tropomyosin alpha-1 chain	33	TPM1_1	95	38	n.d.	491.1±97.8	n.d.	1142.9±535.1
				TPM1_2	208	38	(1497.9±121.8)*	(372.4±15.4)**	851.0±121.8	(1423.1±112.2)*
				TPM1_3	327	37	(407.5±75.3)*	(634.3±71.3)*	n.d.	(3869.8±111.6)**
				TPM1_4	535	37	1525.8±146.5	n.d.	1627.1±91.2	2425.1±39.4
				TPM1_5	409	35	n.d.	n.d.	n.d.	6174.7±731.1
				TPM1_6	407	35	n.d.	n.d.	n.d.	1626.2±624.5
Regulation of signal transduction (GO: 0009966)	PEBP1/PEBP1	Phosphatidylethanolamine-binding protein 1	21	PEBP1_1	241	23	n.d.	426.5±61.5	n.d.	n.d.
				PEBP1_2	148	21	(301.7±12.2)*	(96.0±9.1)**	(350.1±50.1)*	n.d.
Vesicle-mediated transport (GO: 0016192)	ALBU/ALB	Albumin	66	ALB_1	137	22	n.d.	n.d.	263.4±113.4	n.d.
				ALB_2	483	31	n.d.	n.d.	n.d.	195.0±113.4
				ALB_3	260	46	n.d.	n.d.	n.d.	295.8±14.4
				ALB_4	149	44	n.d.	n.d.	n.d.	358.9±35.4
	PGBM/HSPG2	Basement membrane-specific heparan sulfate proteoglycan core protein	398	HSPG2	37	16	n.d.	67.5±12.2	n.d.	206.4±17.1
	HPT/HP	Haptoglobin	38	HP	125	45	249.4±34.8	n.d.	284.7±213.2	n.d.

^a^Biological process(es) according to Gene Ontology (GO) (*16*). ^b^Unique alphanumeric identifier of each entry in SwissProt database (*15*). ^c^Protein name recommended by the SwissProt database consortium (*15*). ^d^Spot on 2-DE gels (Fig. 1). ^e^Ion score is -10∙log(P), where P is the probability that the observed match is a random event. Individual ion scores >35. ^f^Densitometry values (Intensity×spot area/10 000) calculated for specific spot. Significant fold change cut-off was >3.0, and is indicated with stars. n.d.=not detected. * and ** in a row represent statistically significant differences among products at p<0.05 obtained by one-way ANOVA and LSD test

Peptidomic analysis revealed that traditional kulenova seka contained the lowest number of peptides (in total 43), whereas the highest number of peptides was identified in industrial kulenova seka (in total 107). A total of 72 peptides were identified in traditional baranjski kulen and 96 in industrial baranjski kulen. Table 2 shows the list of proteins from which the identified peptide sequences originated. Most of the peptides in all samples arise from actin.

**Table 2:** Identified peptides detected in traditional and industrial baranjski kulen and kulenova seka by LC-MALDI-TOF/TOF mass spectrometry analysis

Biological process^a^	Accession no.^b^/gene	Protein name^c^	Kulenova seka		Baranjski kulen	
Industrial	Traditional	Industrial	Traditional
Peptide no./protein coverage^d^			
Muscle contraction (GO: 0006936)	MYH1/MYH1	Myosin-1	n.d.	n.d.	4/2.2	n.d.
	MYH4/MYH4	Myosin-4	12/1.8	n.d.	n.d.	n.d.
	MYL1/MYL1	Myosin light chain 1/3, skeletal muscle isoform	n.d.	n.d.	2/10.9	5/13.5
	MLRS/MYLPF	Myosin regulatory light chain 2, skeletal muscle isoform	4/7.1	n.d.	7/14.1	5/14.1
	MYG/MB	Myoglobin	n.d.	1/0.4	n.d.	n.d.
	TITIN/TTN	Titin	n.d.	n.d.	1/0.1	n.d.
	TNNI2/TNNI2	Troponin I, fast skeletal muscle	13/18.1	n.d.	3/8.2	n.d.
	TNNT3/TNNT3	Troponin T, fast skeletal muscle	9/15.1	n.d.	9/13.3	7/12.5
	ACTS/ACTA1	Actin, alpha skeletal muscle	55/30.0	28/15.9	51/28.9	49/26.3
Actin filament-based process (GO: 0030029)	STAR8/STARD8	StAR-related lipid transfer protein 8	n.d.	n.d.	1/1.8	n.d.
Small molecule metabolic process (GO: 0044281)	CAH3/CA3	Carbonic anhydrase 3	2/8.5	n.d.	2/7.7	n.d.
	ENOB/ENO3	Beta-enolase	n.d.	n.d.	3/2.5	3/2.5
	KCRM/CKM	Creatine kinase M-type	6/13.6	6/13.9	9/13.6	3/6.0
Cell surface receptor signaling pathway (GO: 0007166)	GP115/ADGRF4	Adhesion G protein-coupled receptor F4	n.d.	n.d.	2 / 5.0	n.d.
Adaptive immune response (GO: 0002250)	PEPL/Ppl	Periplakin	n.d.	n.d.	1/0.4	n.d.
Vesicle-mediated transport (GO: 0016192)	ALBU/ALB	Albumin	n.d.	8/15.3	n.d.	n.d.
	HBA/HBA1	Haemoglobin subunit alpha	1/10.6	n.d.	n.d.	n.d.
Sarcomere organization (GO: 0045214)	LDB3/LDB3	LIM domain-binding protein 3	3/1.4	n.d.	1/1.2	n.d.

^a^Biological process(es) according to Gene Ontology (GO) (*16*). ^b^Unique alphanumeric identifier of each entry in SwissProt database (*15*). ^c^Protein name recommended by the SwissProt database consortium (*15*). ^d^Total number of unique peptides detected in two technical repetitions and protein sequence coverage (%). n.d.=not detected

With respect to the observed proteomic and peptidomic results, according to GO (*16*), most of the identified proteins and peptides can be grouped into: proteins related to muscle contraction, small molecule metabolic process, cellular response to stress and vesicle-mediated transport.

#### Muscle contraction proteins

The most observable protein degradation process affected proteins involved in muscle contraction: myosin heavy chains (myosin-1 and -4), myosin light chain 1/3 (skeletal muscle isoform), titin, troponin I, troponin T and tropomyosin alpha-1 chain. Cathepsins, which can penetrate the myofibrillar structure, are responsible for the initial hydrolysis of myosin during dry-curing (*5*). After the initial myofibrillar protein breakdown by muscle proteinases, the enhanced proteolysis of myofibrillar proteins can be the result of the addition of starter cultures (*7*). Proteolysis of myosin-1 generates large amounts of small peptides in fermented meat products (*21*). Interestingly, peptides originating from myosin-1 were detected only in industrial baranjski kulen. As peptides originating from myosin-1 were not detected in other products, we can assume that a less intense degradation might have occurred in other products. Furthermore, our preliminary results indicate that the intense hydrolysis of myosin-4 occurred in industrial kulenova seka as 12 peptides originating from this protein were detected. It was previously described that titin undergoes intense degradation throughout the processing of dry-cured meat products, generating large amounts of peptides due to the huge size (*22*). In the industrially produced kulenova seka and baranjski kulen partial degradation of titin was detected (Fig. 1). Fragments with estimated molecular masses of 22 and 25 kDa were detected in traditional baranjski kulen, while fragments with a molecular mass of 38 kDa were detected in industrial kulenova seka. Only one peptide originating from titin was identified by peptidomic analysis in industrial kulenova seka. Based on the given results, we can emphasize that titin was not subjected to an intense proteolysis during industrial processing, but to a slow and product type specific degradation. Previous studies have reported low degradation of the myosin light chain during curing in dry sausages compared to fresh meat (*23*). On the contrary, the intense proteolysis of the myosin light chain after the addition of starter cultures in beaker sausages was detected (*7*). Our results showed that an intact myosin light chain 1/3 was present only in traditional kulenova seka, and the corresponding peptides were detected in traditional baranjski kulen. Therefore, we can suppose that a specific degradation occurred during the traditional processing period. The described degradation could be due to different production times. Fadda *et al*. (*23*) also detected myosin light chain 1/3 degradation that may be related to a longer ripening period or to the applied technology.

Cathepsins are also responsible for troponin I hydrolysis during dry-curing (*5*). Significant occurrence of the peptides originating from troponin I has been observed in more tender meat (*24*). Our results indicate partial degradation of troponin I in all products as fragments corresponding to 16 kDa were detected. Furthermore, in traditionally produced baranjski kulen and kulenova seka, fragments with a molecular mass of 14 kDa were also detected. On the contrary, small molecular mass peptides were detected in industrially produced baranjski kulen and kulenova seka, indicating a more intense and processing type-specific degradation process. More intense proteolysis could be the result of the addition of starter cultures to industrial products.

Peptides originating from myoglobin were detected as a result of indigenous muscle proteinase degradation throughout the ripening of fermented sausages (*4*). Furthermore, it was evidenced that genetics can also influence myoglobin degradation during dry-curing of ham (*25*). In our study, a peptide analysis revealed that peptides originated from myoglobin only in traditional kulenova seka. Myoglobin degradation was reported in semi-dry fermented sausages (*4*) and in the beaker sausage model with the addition of autochthonous starter cultures (*7*).

#### Small molecule metabolic process

The protein degradation also affected the proteins included in the small molecule metabolic process: fructose-bisphosphate aldolase A, carbonic anhydrase, aminoacylase-1, isocitrate dehydrogenase [NADP], pyruvate kinase, dihydropyrimidine dehydrogenase [NADP(+)] and beta-enolase. Intact fructose-bisphosphate aldolase A and carbonic anhydrase 3 proteins were detected on 2-DE gels only in traditional baranjski kulen. For both enzymes, fructose-bisphosphate aldolase A and carbonic anhydrase 3 resistance to endogenous and microbial enzyme activity was reported (*26*). Thus, a higher expression level of fructose-bisphosphate aldolase A and carbonic anhydrase 3 in traditional baranjski kulen could be explained by the generally higher level of these proteins in raw meat. However, peptide analysis results showed that peptides originating from carbonic anhydrase 3 were present in other products, industrial baranjski kulen and kulenova seka. These results indicate that carbonic anhydrase 3 underwent proteolysis in other products despite the described resistance. Therefore, in industrial products (baranjski kulen and kulenova seka), the lower level of carbonic anhydrase 3 was more likely related to enhanced proteolysis in industrial products due to the addition of starter cultures than to raw meat composition. Furthermore, our results confirmed partial degradation of pyruvate kinase in all four products. As shown in Fig. 1, fragments were predominantly detected in traditional products, while an intact molecule was confirmed in industrial baranjski kulen. Peptides originating from pyruvate kinase were not detected by peptidomic analysis (Table 2), confirming the partial digestion process. Beta-enolase hydrolysis by endopeptidases and exopeptidases was identified in dry-cured meat such as dry-cured ham and dry-fermented sausages (*25*). Our results pointed out specific degradation of beta-enolase in baranjski kulen products (both industrial and traditional), as peptides originating from beta-enolase were detected only in the baranjski kulen products (Table 2). Intact malate dehydrogenase was identified on 2-DE gels of traditional products (both kulenova seka and baranjski kulen) (Fig. 1). Rosa *et al.* (*24*) demonstrated that malate dehydrogenase expression could be a result of a variety in genotypes. A greater abundance of dihydropyrimidine dehydrogenase [NADP(+)] was found in traditional than industrial kulenova seka, while it was not detected in baranjski kulen. To the best of our knowledge, no information about the degradation of dihydropyrimidine dehydrogenase [NADP(+)] for fermented sausages is available in literature.

#### Cellular response to stress

Proteins involved in cellular response to stress serotransferrin and glutathione S-transferase P were identified in industrial baranjski kulen, while annexin A6 was identified in both industrial and traditional baranjski kulen. The overabundance of these proteins in industrial baranjski kulen could originate from the differences in raw muscle composition. However, it could also be due to the proteolysis or changes in the solubility of protein during denaturation and/or degradation.

#### Vesicle-mediated transport

Albumin, haemoglobin subunit alpha, basement membrane-specific heparan sulfate proteoglycan core protein and haptoglobin are proteins with a role in vesicle-mediated transport. The presence of albumin and haemoglobin subunit alpha in meat is probably related to the remaining blood in the muscles after bleeding and it is commonly identified in the muscles (*27*). The peptide analysis revealed the peptides originating from albumin specific for traditional kulenova seka, while partial digestion of serum albumin was detected on 2-DE gels of baranjski kulen. Our results indicate that a different proteolysis process occurred in baranjski kulen products compared to kulenova seka. This could be due to different calibre and different production duration. Piñeiro *et al.* (*28*) showed that the concentration of haptoglobin increased significantly from a base level as a result of animal tissue inflammation. Detection of haptoglobin only in industrial products could point to the better health of animals used in traditional production.

### Amino acids and aroma profiles

**Fig. 2:**
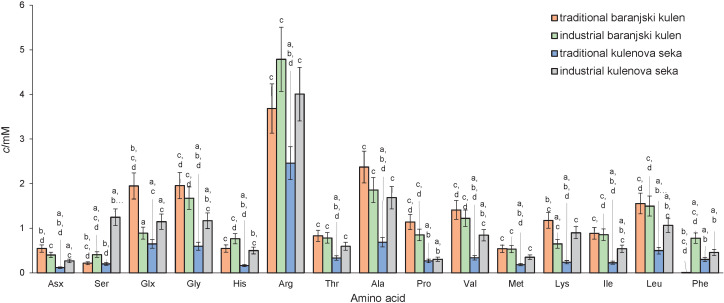
Free amino acid composition of industrial and traditional baranjski kulen and kulenova seka. Error bars represent the standard deviation for each product. Different letters represent statistically significant differences among products. Asx=aspartic acid and asparagine, Ser=serine, Glx= glutamic acid and glutamine, Gly=glycine, His=histidine, Arg=arginine, Thr=threonine, Ala=alanine, Pro=proline, Val=valine, Met=methionine, Lys=lysine, Ile=isoleucine, Leu=leucine and Phe=phenylalanine

Quantitative comparison of the concentration of the amino acids between traditional and industrial baranjski kulen and kulenova seka is shown in Fig. 2. From the amino acid profile, it can be observed that significantly lower concentrations of almost all amino acids were measured in traditional kulenova seka than in other products. These results are in correlation with the peptide analysis and could be related to lower protein degradation. However, the low amount of amino acids after fermentation could also indicate their metabolisation by bacteria (*2*). The predominant amino acids in all samples were arginine and glutamate/glutamine. A similar profile was found in Spanish sausage, where the highest amounts of glutamine, arginine and tyrosine were measured (*22*). A high concentration of glutamic acid was also detected in the sausages after the addition of starter cultures (*2*). During drying and ripening of other types of sausages, an increase in the concentration of phenylalanine was observed (*29*). On the contrary, in traditional baranjski kulen, very low concentrations of phenylalanine were measured compared to other products. This may be due to the drying and ripening processes (*29*), but also due to amino acid degradation pathway (*30*).

Relative amounts of volatile aroma compounds detected in the headspace of baranjski kulen and kulenova seka produced by traditional and industrial technologies are reported in Table 3. In total, 43 compounds were (tentatively) identified, including four terpenes, two organosulphur compounds, three miscellaneous compounds, 11 benzenoids, 19 phenols and 4 furanoid and pyranoid compounds.

**Table 3:** Relative amounts of peak area (*A*_peak_) of volatile compounds in industrial and traditional baranjski kulen and kulenova seka

Volatile compound	*A*_peak_/10 000					
Baranjski kulen			Kulenova seka	
Industrial	Traditional		Industrial	Traditional
Terpenes					
Limonene	9.2±8.6	112.5±45.7		n.d.	40.9±15.8
Terpene n.i. 121, 93, 161	32.6±1.0	72.3±47.8		32.6±1.9	30.8±11.8
Terpene n.i. 133, 93, 161 (caryophyllene structure)	137.0±21.2	205.6±154.1		123.1±10.4	94.2±27.6
Terpene n.i. 93, 121, 147 (humulene structure)	18.5±3.2	24.6±18.9		19.0±3.1	7.6±7.7
					
Organosulfur compounds					
Methyl ethyl sulphide	2.0±2.8	n.d.		3.7±2.2	n.d.
Diallyl disulphide	16.9±13.7	13.3±3.5		20.0±10.7	44.5±19.1
					
Miscellaneous					
Heptanal	1.5±1.7	0.4±0.6		0.8±0.3	n.d.
Methyl 2,4-hexadienoate	n.d.	n.d.		50.4±11.9	n.d.
*n*-Hexadecanoic acid	5.2±4.9	50.7±43.8		1.8±0.8	63.8±90.9
					
Benzenoids					
Phenylmethanal	1132.3±1201.3	375.7±389.4		421.1±131.4	105.0±48.2
Phenyl cyanide	n.d.	(68.8±76.1)^b^		n.d.	(513.9±39.4)^a^
Phenylmethanol	(110.1±61.6)^bc^	(177.7±30.9)^b^		(322.3±34.1)^a^	(31.9±9.7)^c^
Phenylacetaldehyde	(10895.9±6451.7)^a^	(1522.5±599.8)^b^		(1163.3±545.2)^b^	(102.4±64.2)^b^
Phenylethanol	353.6±465.0	58.0±14.5		83.8±32.9	64.5±26.1
3,4-Dimethoxytoluene	103.5±45.9	283.7±126.3		92.2±13.8	270.7±153.0
1,4-Dimethoxy-2-methylbenzene	63.4±29.5	143.4±87.7		85.6±13.3	133.1±99.4
Indane-4-carboxaldehyde	51.6±32.4	n.d.		n.d.	n.d.
1,2,3-Trimethoxybenzene	17.5±7.6	74.2±63.9		19.2±4.6	46.4±31.5
Acetyl eugenol	136.4±89.9	82.7±81.6		173.3±79.6	141.8±129.2
1,2,4-Trimethoxybenzene	127.3±57.6	107.6±76.2		192.6±86.9	146.4±70.5
					
Phenols					
Phenol	(177.9±162.8)^b^	(411.2±279.7)^b^		(628.2±71.7)^b^	(1487.5±232.6)^a^
2-Methylphenol	(204.9±48.9)^b^	(338.2± 99.6)^b^		(401.3±119.7)^b^	(718.4±109.5)^a^
3-Methylphenol	(262.0±117.3)^b^	(547.0±48.6)^b^		(508.4±147.5)^b^	(1401.9±396.9)^a^
2-Methoxyphenol	(1811.9±1049.6)^b^	(1630.4±989.6)^b^		(3104.7±626.1)^b^	(5299.3±615.9)^a^
2,6-Dimethylphenol	44.1±19.9	68.3±11.8		63.9±17.8	96.5±31.8
2,4-Dimethylphenol	125.4±75.4	306.8±118.4		158.1±65.6	445.2±257.8
4-Ethylphenol	14.6±13.4	212.6±123.6		28.2±13.8	271.6±162.3
3-Ethylphenol	14.6±6.8	65.4±29.4		23.2±10.1	91.7±53.3
3,5-Dimethylphenol	32.2±17.3	116.0±70.4		42.4±16.9	141.2±84.1
2,3-Dimethylphenol	n.d.	48.0±23.7		19.0±4.9	56.8±36.8
4-Methoxy-3-methylphenol	(129.8±98.4)^b^	(208.4±78.0)^b^		(245.9±55.0)^b^	(422.7±45.5)^a^
2-Methoxy-4-methylphenol	(1466.3±691.3)^b^	(1498.8±152.3)^b^		(2083.1±505.5)^b^	(3489.9±747.6)^a^
2,4,5-Trimethylphenol	n.d.	n.d.		n.d.	25.6±25.3
4-Ethyl-2-methoxyphenol	557.5±287.8)	2274.1±1316.6		771.6±242.7	2657.6±1371.2
2-Methoxy-4-vinylphenol	21.8±11.3	56.1±34.0		84.3±63.5	200.4±102.7
2-Methoxy-4-propylphenol	74.8±43.1	1067.2±920.8		97.6±36.2	518.2±532.1
Eugenol	83.0±45.2	92.0±77.5		107.4±40.6	103.5±79.3
2,6-Dimethoxyphenol	351.5±143.3	278.1±142.7		487.9±182.1	536.2±284.2
*trans*-Isoeugenol	29.3±19.4	57.6±57.6		38.7±19.0	48.3±42.6
					
Furanoids and pyranoids					
3-Ethenylpyridine	n.d.	n.d.		n.d.	184.2±46.5
Benzofuran	n.d.	86.6±95.7		25.3±8.1	181.0±104.9
2,4-Dimethylfuran	(56.9±52.2)^b^	(49.7±70.3)^b^		(115.1±3.4)^ab^	(184.9±30.2)^a^
2-Acetyl-5-methylfuran	n.d.	n.d.		n.d.	145.9±33.4

Different lowercase letters in a row represent statistically significant differences among products at p<0.05 obtained by one-way ANOVA and LSD test

Only a few terpenes were detected in relatively low amounts (Table 3). Terpenes in dried meat products are found mainly as ingredients of added spices (*17*). Since the production of baranjski kulen and kulenova seka did not involve spices abundant in monoterpenes, the obtained lower amounts were expected. Similar results were obtained previously for slavonski kulen (*31*). The identified terpenes possibly originated from black and ground red pepper used in the production, since these spices contain moderate amounts of terpenes (*17*). The identified terpenes may also originate from the animal feed (*32*). In this study, no significant differences in terpene amounts were found among the investigated products.

Organosulphur compounds identified in this study (Table 3) most certainly originated from garlic, used as a spice in the production of baranjski kulen and kulenova seka. These compounds are produced after disruption of garlic bulb tissues, which activates the allinase enzyme to produce allicin from alliin. Allicin is then further decomposed to finally yield volatile sulphur compounds with a general formula R_1_–(S)_n_–R_2_. Among them, diallyl disulphide, probably a direct product of allicin degradation (*33*), is the most abundant volatile compound in garlic (*34*). Consequently, it is the most representative organosulphur compound in the volatile profile of slavonski kulen, as shown previously by Jerković *et al*. (*31*) and confirmed in this study with baranjski kulen and kulenova seka. No significant difference in the amount of diallyl disulphide was determined between industrial and traditional baranjski kulen and kulenova seka, respectively (Table 3). However, another organosulphur compound, methyl ethyl sulphide, was detected only in industrial products. In addition, volatiles derived from spices, especially compounds originating from garlic, can be subjected to oxidative degradation due to increased amounts of unsaturated fatty acids. Also, the differences between samples could originate from the differences in raw material (*35*). Since these parameters were not controlled in this study, their effects should be investigated in detail in the future.

Interestingly, compounds generated in the lipid degradation processes including autooxidation, mostly represented by short-chain saturated and unsaturated aldehydes, ketones and alcohols that contribute to the aroma of a sausage with rancid or oxidised sensory attributes, were barely detected. Such a result corresponded to that obtained by Jerković *et al*. (*31*), who found relatively low percentages of these compounds in the volatile profile of slavonski kulen. The authors related this to the occurrence of high concentrations of phenols and furans, which are derived from the smoking applied during curing (*36*, *37*). They act as antioxidants and therefore limit autoxidation. In this study, a higher level of heptanal was found in both industrial than in both traditional sausages. On the contrary, higher amount of *n*-hexadecanoic acid was found in traditional baranjski kulen and kulenova seka. It is possible that both compounds derived from lipolysis, with the increased amount of *n*-hexadecanoic acid in traditional sausage, probably as a direct result of increased triglyceride hydrolysis (*38*), and the increased amount of heptanal in industrial sausage as a consequence of the greater activity of other enzymes.

Compounds from the amino acid degradation pathway are mainly represented by phenylalanine degradation products (*30*), such as phenylmethanol, phenylmethanal, phenylacetaldehyde, phenyl cyanide and phenylethanol. Industrial baranjski kulen was the most abundant in phenylacetaldehyde, while industrial kulenova seka contained the highest amount of phenylmethanol. Methoxylated benzenoids identified in this study were also possibly partly derived from the oxidative and/or non-oxidative degradation of l-phenylalanine (*39*), however, no significant differences were observed among the products regarding their amounts. The occurrence of indane-4-carboxaldehyde was specific for industrial baranjski kulen. A tendency towards higher amounts of benzene-based compounds, such as 3,4-dimethoxytoluene, 1,4-dimethoxy-2-methylbenzene, 1,2,3-trimethoxybenzene, although without statistical significance, was noted in traditional products. These compounds are known to originate from smoke used in the production of smoke-cured meat products (*40*).

Benzonitrile was found only in industrial products. It is known that this and similar compounds, such as phenylacetonitrile, derive from the reaction of acids and sodium nitrite. As production of industrial baranjski kulen and kulenova seka included the addition of nitrites, preservatives added to inhibit the growth of bacteria (*41*), benzonitrile identification was not surprising.

Qualitatively, the most abundant group of compounds identified in the industrial and traditional baranjski kulen and kulenova seka investigated in this study were smoke-derived volatile phenols (Table 3). Among them, methoxyphenols, such as 2-methoxyphenol and 4-ethyl-2-methoxyphenol, dominated the profile. This was slightly in disagreement with the results of Jerković *et al.* (*31*), who found higher concentrations of phenol and certain methyl-substituted phenols than methoxyphenols in the volatile profile of slavonski kulen. However, the amounts of the former compounds in this study were also high. The highest amounts of phenol, 2-methylphenol, 3-methylphenol, 2-methoxyphenol, 4-methoxy-3-methylphenol and 2-methoxy-4-methylphenol were found in traditional kulenova seka. The 2,4,5-trimethylphenol was detected only in this product. Besides, a tendency towards higher amounts of particular ethyl, vinyl and propyl phenols was observed in traditional products, although without a significant difference (Table 3). It is possible that the traditional process included a higher degree of smoking than the industrial one or that the differences derived from different types of wood used. Another source of volatile phenols is enzymatic degradation by microorganisms (*42*), so higher amounts in traditional products could be connected to the use of smaller amounts of preservatives in their production, as suggested above. According to other authors (*31*, *42*), smoke-derived phenols exhibit low sensory threshold values that make them important contributors to slavonski kulen flavour with their woody, pungent and smoky odours.

Traditionally produced kulenova seka was characterized by the highest amount of 2,4-dimethylfuran. Benzofuran also exhibited a tendency towards the highest values in this product, while two other compounds from this group, 3-ethenylpyridine and 2-acetyl-5-methylfuran, were detected only in traditional kulenova seka (Table 3). Similarly to phenols, furans are also characteristic of wood smoke derived from cellulose, hemicellulose and lignin pyrolysis, originating mainly from the Maillard reaction (*43*). During smoking, these compounds are adsorbed by the sausage surface and a part of them interacts with other sausage components. A fraction that remains adsorbed on the surface but does not change chemically by the mentioned interactions is present in its headspace and contributes to the smoked sausage flavour (*31*). It is possible that a higher degree of smoking in the production of traditional sausage in this study was the main cause of the observed differences.

## CONCLUSIONS

A comprehensive proteome and metabolome analysis provided the first insight into the molecular differences between industrially and traditionally prepared fermented sausages baranjski kulen and kulenova seka, including the differences in protein and peptide profiles, free amino acid content and aroma profiles. The results pointed to potential product type-specific quality markers for each sausage: industrial baranjski kulen – peptides originated from myosin-1 and titin, overabundance of stress-related proteins serotransferrin, glutathione S-transferase P and annexin, and the highest amount of phenylalanine; traditional baranjski kulen - low concentration of phenylalanine and overabundance of fructose-bisphosphate aldolase A and carbonic anhydrase 3; industrial kulenova seka - specific myosin-4 and haemoglobin subunit alpha degradation process; traditional kulenova seka - overabundance of dihydropyrimidine dehydrogenase [NADP(+)] and myosin light chain 1/3, skeletal muscle isoform, degradation of albumin and myoglobin, low concentrations of almost all free amino acids and increased amounts of smoke-derived volatile compounds. The main differences between industrial and traditional products are an enhanced proteolytic degradation in industrial products, detected haptoglobin, as well as benzonitrile and higher amounts of heptanal. The presented results provide important information in overcoming the challenges of the meat industry in obtaining a final product with the characteristics of a traditional product.
